# Complete chloroplast genome of an orchid species *Cymbidium floribundum* Lindl

**DOI:** 10.1080/23802359.2019.1662743

**Published:** 2019-09-06

**Authors:** Guo-Qiang Zhang, Zhong-Jian Liu, Si-Ren Lan

**Affiliations:** a Forestry College of Fujian Agriculture and Forestry University, Fuzhou, China;; b Key Laboratory of National Forestry and Grassland Administration for Orchid Conservation and Utilization, Shenzhen Key Laboratory for Orchid Conservation and Utilization, The National Orchid Conservation Center of China and The Orchid Conservation and Research Center of Shenzhen, Shenzhen, China;; c Key Laboratory of National Forestry and Grassland Administration for Orchid Conservation and Utilization, Fuzhou, China

**Keywords:** *Cymbidium floribundum*, chloroplast genome, phylogenetic, Orchidaceae

## Abstract

*Cymbidium floribundum* Lindl. is an ornamental plant and its native range is south China to north Vietnam. Here, we report the complete chloroplast (cp) genome sequence and the cp genome features of *C. floribundum*, which was assembled and characterized based on Illumina pair-end sequencing data. The complete chloroplast genome was 153998 bp in length. Its structure contained a large single-copy (LSC) region of 84,725 bp and a small single-copy (SSC) region of 19,009 bp, which were separated by a pair of extremely inverted repeats (IRs) of 25,132 bp each. The phylogenetic analysis indicated that *C. floribundum* was in the basal of subgenus *Jenson*.

The genus *Cymbidium*, with approximately 80 species that are primarily distributed throughout the subtropics and tropical areas of Asia and the northern Australia (Chen et al. 2009; Pridgeon et al. 2009). China is one of the distribution centre of *Cymbidium* with over 50 species, of which 19 are endemic (Liu et al. 2006; Chen et al. 2009; 2019; Zhang et al. 2019). *Cymbidium floribundum* Lindl. is an ornamental plant belonging to this genus (Chen et al. 2009). However, in spite of its ecological and ornamental value, genomic studies have been hindered due to lack of information about the complete chloroplast (cp) genome of *C. floribundum*. In the present study, the complete chloroplast genome sequence of *C. floribundum* was assembled to improve an appreciation of its genomics.

Leaves were collected from a single individual of *C. floribundum* at the Orchid Conservation and Research Centre of Shenzhen and specimens were deposited in the National Orchid Conservation Center herbarium (NOCC; specimen code Z.J.Liu 3256). Total genomic DNA was extracted from fresh material using the modified CTAB procedure of Doyle and Doyle (1987). Sequenced on Illumina Hiseq 2500 platform (San Diego, CA, USA). Genome sequences were screened out and assembled with MITObim v1.8 (Hahn et al. 2013), which resulted in a complete circular sequence of 153,998 bp in length. The cp-genome was annotated with CpGAVAS (Liu et al. 2012).

The whole cp genome was 153,998 bp in length (GenBank accession MK848043), containing a pair of inverted repeats (IRs) of 25,132 bp each, a large single-copy region (LSC, 84,725 bp), and a small single-copy region (SSC, 19,009 bp). The cp genome encoded 130 genes, of which 107 were unique genes (80 protein-coding genes, 23 tRNAs and 4 rRNAs).

In order to investigate the phylogenetic status of *C. floribundum*, the available complete cp genomes of 17 species were aligned using MAFFT (Katoh and Standley 2013) with the default parameters. A maximum likelihood (ML) analysis was reconstructed from all of the 17 complete cp genome sequences by the CIPRES Science Gateway webserver (RAxML-HPC2 on XSEDE 8.2.10) with 1000 bootstrap replicates and settings as described by Stamatakis et al. (2008). The result of a phylogenetic analysis indicated that *C. floribundum* was in the basal of subgenus *Jenson* (Figure 1). Furthermore, the complete cp genome of *C. floribundum* will provide useful genomic information for detailed population genetic studies in the future.

**Figure 1.**
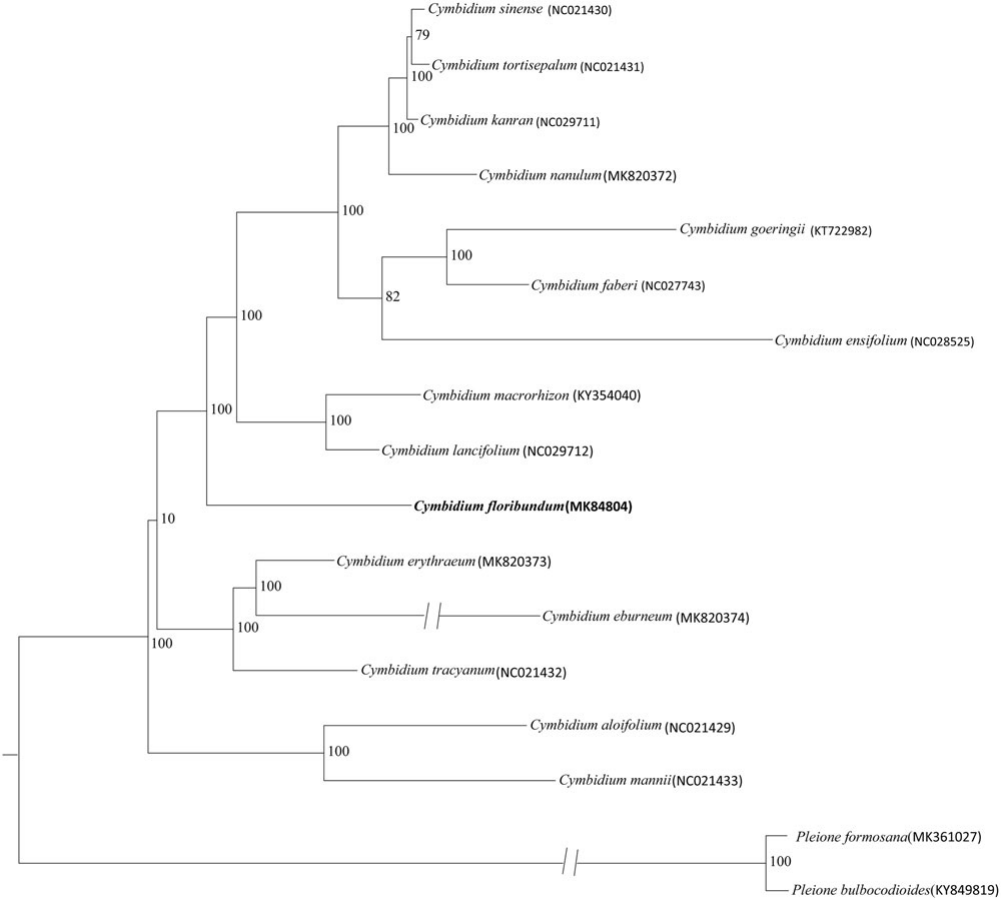
Phylogenetic position of *Cymbidium floribundum* inferred by maximum likelihood (ML) of complete cp genome. The bootstrap values are shown next to the nodes.
